# PDMS/Ag/Mxene/Polyurethane Conductive Yarn as a Highly Reliable and Stretchable Strain Sensor for Human Motion Monitoring

**DOI:** 10.3390/polym14245401

**Published:** 2022-12-09

**Authors:** Shichen Zhang, Jiangtao Xu

**Affiliations:** 1 School of Innovation Design, Guangzhou Academy of Fine Arts, Guangzhou 510006, China; 2 College of Materials and Energy, South China Agricultural University, Guangzhou 510006, China

**Keywords:** protective layer, strain sensor, yarn, human motion, monitoring

## Abstract

The conductivity and sensing stability of yarn-based strain sensors are still challenges when it comes to practical applications. To address these challenges, surface engineering of polyurethane (PU) yarn was introduced to improve its surface hydrophilicity for better deposition of MXene nanosheets in its dispersion. The introduction of Ag nanoparticles via magnetron sputtering greatly improved the surface conductivity; meanwhile, the encapsulation of the PDMS protective layer effectively enhanced the sensing stability over 15,000 cycling process, as well as the working range with a gauge factor value over 700 under a strain range of 150–300%. Moreover, the exploration of its applications in human motion monitoring indicate that the prepared strain-sensing yarn shows great potential in detecting both tiny motions or large-scale movements of the human body, which will be suitable for further development into multifunctional smart wearable sensors or metaverse applications in the future.

## 1. Introduction

Recently, wearable and flexible electronic devices have been widely used in human motion monitoring [1,2], man–machine interactive systems [3,4], or healthcare monitoring [5,6], by providing useful information for treatment options. Among the emerged strain sensors, fiber-based strain sensors may be ideal for multiscenario applications due to their advantages. These kinds of strain sensors not only show characteristics of light weight, good mechanical performance, and small size, but also present excellent implantability, such as being embedded into other fabrics or woven into electronic textiles [7,8]. Therefore, they are ideal strain sensors for the next generation of wearable electronic devices.

Nowadays, the most favorable and effective method for fabricating yarn- or fiber-based strain sensors is preparing conductive polymer composite yarns via adding conductive materials, including carbon black [9,10], carbon nanotubes [11,12], graphene [13,14], and so on. Usually, conductive materials are deposited onto the surface of elastic fibers via the dip-coating method, and the conductive materials can be evenly distributed on the surface of elastic substrates, resulting in excellent conductivity and stretchability. Nevertheless, the conductive materials are easily peeled off during the repeated testing cycles due to weak adhesion; this leads to low durability and poor stability, which is not desired for a strain sensor [15,16]. Improving stability and conductivity is still a challenge for current research.

In recent years, the surface/interface engineering method has been a favorable strategy to prepare conductive yarns with high conductivity and stability. For example, He et al. reported that a conductive composite CNT/MXene layer was deposited onto the surface of the polyurethane (PU) yarn via the dip-coating method, and an ultrathin polyurethane layer was employed as a bridge layer to improve the adhesion between the conductive layer and PU yarn to prepare conductive yarn for the strain sensor. The interface interaction between the PU yarn and conductive composite layer was strengthened by the introduced polyurethane thin layer, thereby improving the stability during repeated stretching/releasing tests [17]. Li et al. prepared a PU yarn-based strain sensor with graphene as conductive layer, and the performance was improved by employing poly(vinyl alcohol) as binder to enhance the interaction between graphene and PU yarn during the dip-coating process [18]. Nevertheless, the conductivity of the prepared yarn remains at a high level due to the limited loading area; moreover, the conductive materials are easily peeled off during the test, weaving, or knitting process. This really challenges the development of yarn-based strain sensors.

In the current work, we added another two steps to solve the problems, including poor conductivity and adhesion of the outer layer. Firstly, the PU yarn was pretreated with oxidizing solution to endow the surface with the hydrophilic group, then the treated yarn was further immersed into cetyltrimethylammonium bromide (CTAB) solution to self-assemble a CTAB layer to improve the absorption of conductive materials. MXene (Ti_3_C_2_T_x_), which presents great conductivity with excellent dispersion performance, was employed as the conductive layer. The pretreated yarn was coated with MXene for 20 cycles via the dip-coating method. To further improve its conductivity, magnetron sputtering was applied to deposit silver nanoparticles on the surface of MXene@PU yarn. To prevent the peeling-off of conductive materials, the yarn was dipped into a diluted polydimethylsiloxane (PDMS) solution to form a protective layer. Therefore, the prepared yarn presented enhanced electrical and mechanical performance, including conductivity, working range, and stability. Furthermore, the prepared conductive yarn was also applied into the monitoring of physiological signals, such as finger or joint movement and posture detection.

## 2. Experimental

### 2.1. Materials

Ti_3_AlC_2_ powder was purchased from Xianfeng Nano Materials Technology Co., Ltd. (Nanjing City, China) without any further purification for hydrogel synthesis. PU yarn was purchased from Haining Kaiwei Textile, Ltd. (Haining, China). Hydrochloric acid (HCl), lithium fluoride (LiF), concentrated sulfuric acid, hydrogen peroxide solution, cetyltrimethylammonium bromide, and tetrahydrofuran were purchased from Sigma Aldrich. Vinyl-terminated base and curing agent for Polydimethylsiloxane were purchased from Dow Corning (Sylgard^®^ 184).

### 2.2. Synthesis of Mxene (Ti_3_C_2_T_x_)

The MXene was synthesized according to previous report [19]. In detail, 1 g Ti_3_AlC_2_ powder was slowly added into a mixed solution containing HCl (6 M, 10 mL) and LiF (0.666 g), and stirred at a speed of 250 rpm for 24 h at 35 °C. Then, the obtained suspension was washed with deionized (DI) water and centrifuged at 3500 rpm multiple times until the pH reached about 6. The final obtained suspension was ultrasonically treated in an ice bath for 1 h to obtain Ti_3_C_2_T_x_ nanosheet dispersion with a concentration of 2 mg/mL.

### 2.3. Pretreatment of PU Yarn

PU yarn lacks a hydrophilic group, and this is adverse to the following dip-coating process. Therefore, the PU yarn was first treated in a piranha solution for 30 s to endow hydrophilic groups forming on its surface. Then, the treated PU yarn was further immersed into CTAB solutions (0.05 M) for 24 h to form a layer of CTAB on the surface of PU yarn, which will contribute to the absorption of MXene nanosheets to form a conductive layer.

### 2.4. Fabrication of MXene/PU Yarn

The MXene/PU yarn was prepared by using dip-coating method, which is a simple, low-cost, and scalable layer-by-layer assembly method. In detail, the pretreated yarn was immersed into the prepared dispersion of MXene nanosheets for 5 min, then rinsed with DI water and dried in the oven with a temperature of 50 °C for 2 min. The coating procedure was repeated 20 times to produce MXene/PU yarn.

### 2.5. Fabrication of Ag/MXene/PU Yarn

To improve the conductivity of prepared yarn, magnetron sputtering was applied to the deposition of silver nanoparticles on the surface of MXene/PU yarn. In detail, the chamber was firstly pumped into a vacuum of 5 × 10^−4^ Pa, then silver nanoparticles were deposited on the surface of PU yarn with a power of 80 W for 20 min under the activated gas of Argon.

### 2.6. Fabrication of PDMS/Ag/MXene/PU Yarn

To prevent the conductive layer peeling off from PU yarn, a PDMS protective layer was further coated on the surface of prepared conductive yarn. In detail, 0.5 g PDMS was firstly prepared by mixing solution A and B according to a weight ratio of 10:1, then PDMS was diluted by adding 10 mL tetrahydrofuran. Then, the prepared conductive yarns were immersed into the PDMS solutions for 3–5 s and taken out immediately, then dried in an oven at a temperature of 60 °C for 5 h.

### 2.7. Characterization

The surface morphology was obtained via scanning electron microscopy (SEM, TM3000, Hitachi, Tokyo, Japan) and field-emission SEM (Tescan MAIA3; to improve the quality of obtained images, the samples were first fixed on the holder and then coated with gold to improve the conductive contact between samples and the holders). Chemical analysis was confirmed via spectrum 100 spectrometer (PerkinElmer, Waltham, MA, USA). The mechanical performance was tested by using an Instron 5944 MicroTester. The conductivity of prepared conductive yarns was measured via a 4-point probe resistivity meter (ST-2258A, Suzhou Jingge Electronic Co., Ltd., Suzhou, China). The sensing performance was tested by using a combined system including Instron 5944 MicroTester and a Keithley 2010 SourceMeter.

## 3. Results and Discussion

### 3.1. Fabrication of Sensing Conductive Yarns

**Figure 1:**
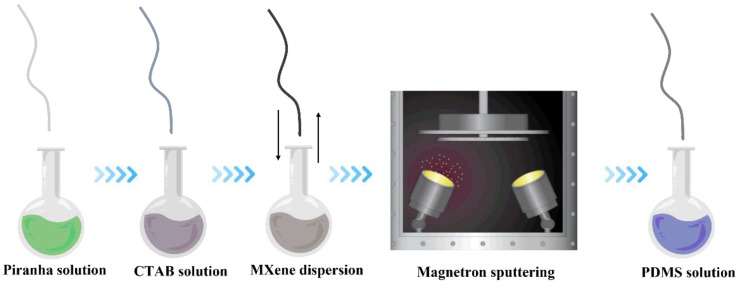
Schematic illustration of fabricating conductive strain-sensing yarns.

**Figure 2:**
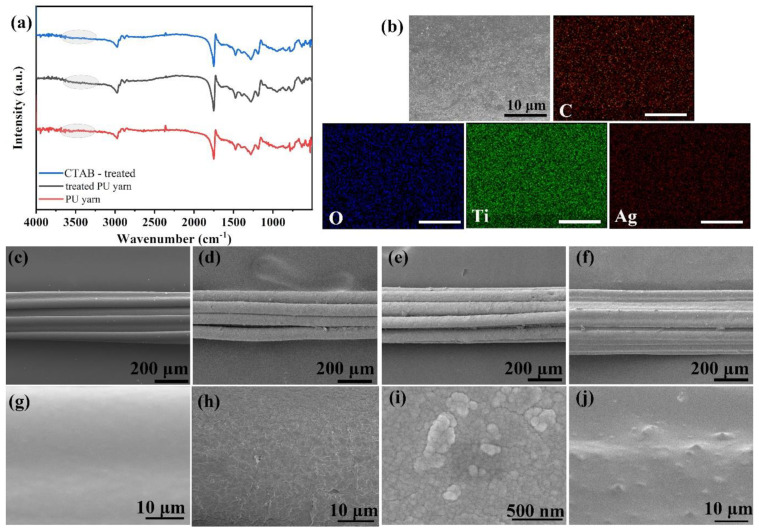
(**a**) FTIR of treated yarns, (**b**) EDX mapping of Ag/MXene/PU yarn (scale bar: 10 μm), (**c**) and (**g**) SEM images of pure PU yarn, (**d**) and (**h**) MXene/PU yarn, (**e**) and (**i**) Ag/MXene/PU yarn, (**f**) and (**j**) PDMS/Ag/MXene/PU yarn.

The fabrication process of conductive strain-sensing yarn was illustrated in Figure 1. The PU yarn was firstly pretreated with piranha solution for 30 s to endow hydrophilic groups onto the surface of the PU yarn. To confirm the formed hydrophilic groups, FTIR spectra were obtained and shown in Figure 2a. The characteristic peaks of PU yarn located at 3324 cm^−1^ (N-H), 2855 cm^−1^ (N-H), 2940 cm^−1^ (-CH), 1730–1706 cm^−1^ (-CO), and 1100 cm^−1^ (C-O-C) were successfully detected in both pure PU yarn and piranha solution-treated PU yarn, indicating that the oxidation treatment did not destroy the structure of PU yarn. It is also observed that the yarn shows enhanced intensity around 3400 cm^−1^ after piranha solution treatment, as marked in Figure 2a; this indicates that the treatment successfully endowed hydrophilic groups on the surface of PU yarn.

To further improve its hydrophilicity, the pretreated PU yarn was immersed into CTAB solution for assembling a layer of CTAB on its surface for better combination of MXene nanosheets in next step. However, the FTIR spectra failed to detect its characteristic peaks. To identify the role of CTAB treatment, conductivities of yarn with different treatments were listed in Table S1 to compare the performance. It can be found that conductivity of yarn with CTAB treatment only is better than that of yarn with piranha solution treatment only. This phenomenon reveals that CTAB can be absorbed on the yarn surface and can contribute to the deposition of MXene nanosheets. Then, the pretreated PU yarn was dip-coated with MXene nanosheet dispersion for 20 times. This can be observed in the SEM images illustrated in Figure 2. The pure PU yarn shows a smooth surface, and after being coated with MXene, the surface becomes coarse. When the magnification is enlarged, tightly stacked sheet material can be observed; this proved that the MXene nanosheets were successfully coated on the PU yarn surface. The conductivity of prepared MXene/PU yarn reached 112 ms/cm, and the conductivity is hard to further improve via the dip-coating method. Therefore, the coated yarn was further deposited with silver nanoparticles through magnetron sputtering. It is observed in Figure 2e,i that there is no obvious appearance change, and silver nanoparticles can be found under a higher magnification field. To further confirm the coated layers, including MXene and Ag nanoparticles, EDX mapping was recorded and shown in Figure 2b. Element C comes from the PU yarn and MXene; the existence of Ti confirms the successful deposition of MXene. Ag is also detected, which confirms that the obtained conductive yarn is Ag/MXene/PU yarn. After magnetron sputtering treatment, the conductivity of the obtained Ag/MXene/PU yarn improved to 441.3 ms/cm.

However, the conductive materials deposited on the elastic substrates are easily peeled off during tests or applications, as mentioned in the Introduction. Therefore, here a PDMS encapsulation method was introduced. To reduce the effect of PDMS on the appearance of conductive yarn and the change in diameter, PDMS was firstly diluted with tetrahydrofuran, then the Ag/MXene/PU yarn was immersed into the solution for 3–5 s and taken out immediately. The optical image (Figure S1) of the yarn before and after PDMS coating treatment also shows little difference. As observed in the SEM images (Figure 2f,j), the surface of the conductive yarn was successfully covered with a thin layer of PDMS.

### 3.2. Mechanical Performance

**Figure 3:**
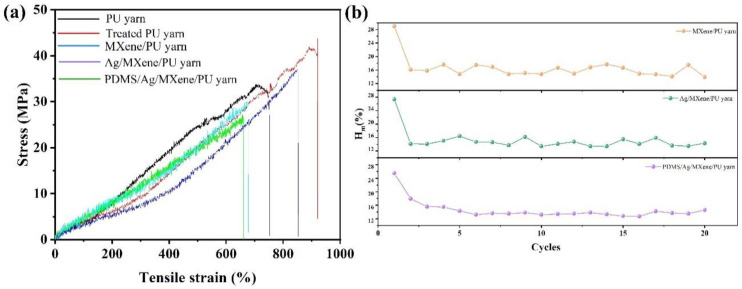
Mechanical performance, (**a**) strain–stress curves of different samples, (**b**) mechanical hysteresis of prepared conductive yarns.

Mechanical performance is a very important basic property for a strain-sensing yarn. Figure 3a shows the stress–strain curves of different samples. The yarn after etching treatment shows a larger tensile strain, which may be caused by the hydrogen bond formed from the endowed hydrophilic groups. After being coated with MXene and silver nanoparticles, there is a reduction in tensile strain performance. This may be caused by the solidification effect from the deposited conductive materials [20]. After PDMS encapsulation treatment, the tensile performance was largely improved due to the introduction of another elastic material (PDMS). Overall, the as-prepared conductive yarns show high tensile strength over 500%, which is preferable for a strain-sensing yarn. The results confirm the excellent mechanical performance of the prepared strain-sensing yarn.

Mechanical hysteresis (*Hm*) is another important parameter that can directly affect the reliability of the prepared strain-sensing yarns. Therefore, the Hm values of different conductive yarns were calculated according to Equation (1) [21,22]:

(1) $$Hm=\frac{\left|As-Ar\right|}{As}$$

where *As* stands for the area under the curves of stretching path, and *Ar* means the area under the curves of releasing path. The stretching/releasing curves (20 cycles) of different conductive yarns were recorded and are presented in Figure S2. After being calculated according to Equation (1), the variations of Hm with different cycles for different conductive yarns were plotted and are shown in Figure 3b. The figure shows that the Hm value of the first cycle of all three conductive yarns are higher than that of after two cycles. The big difference may result from the formed cracks in stretching/releasing process of the first cycle. The average values of Hm after the second cycle were calculated to be 15.67% with an RSD of 1.7% for MXene/PU yarn, 13.68% with an RSD of 1.21% for Ag/MXene/PU yarn, and 13.22% with an RSD of 0.9% for PDMS/Ag/MXene/PU yarn. This result reveals that the prepared strain-sensing yarn, especially PDMS/Ag/MXene/PU yarn, shows excellent reliability.

### 3.3. Electromechanical Performance

**Figure 4:**
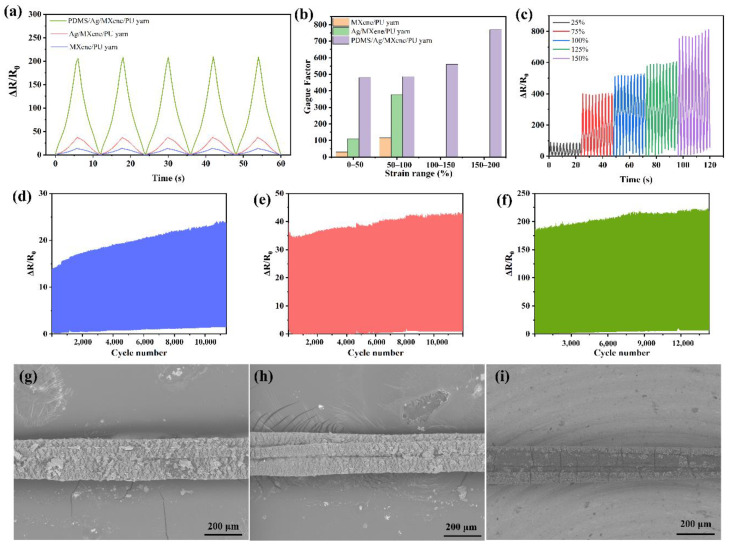
(**a**) Different strain-sensing yarns tested under 5 stretching/releasing cycles, (**b**) gauge factors of different strain-sensing yarns under different strain range, (**c**) signal response of PDMS/Ag/MXene/PU yarn under different tensile ranges, (**d**) cyclability of MXene/PU yarn with an applied strain of 50%, (**e**) cyclability of Ag/MXene/PU yarn with an applied strain of 50%, (**f**) cyclability of PDMS/Ag/MXene/PU yarn with an applied strain of 50%, (**g**) SEM images of MXene/PU yarn with an applied strain of 50%, (**h**) SEM images of Ag/MXene/PU yarn with an applied strain of 50%, (**i**) SEM images of PDMS/Ag/MXene/PU yarn with an applied strain of 50%.

Electromechanical performance is the basic and most important property of strain sensing yarns; therefore, relevant measurements were carried out for evaluation. The responding performances of strain versus resistance for different conductive yarns were recorded and presented in Figure 4a. There is an obvious difference among different conductive yarns, where PDMS/Ag/MXene/PU yarn shows the best signal intensity and Ag/MXene/PU yarn is second, while MXene/PU yarn shows poor performance.

The SEM images illustrated in Figure 4g–i present the stretching state of the three different conductive yarns with an applied strain of 50%. It can be concluded that the different electromechanical performance of the three conductive yarns mainly comes from the different elasticities of conductive layers and substrate. This results in cracks forming on the surface of the elastic yarn with different sizes or shapes when strains are applied. The formation of cracks will lead to the changes in the electrical conductivity of prepared strain-sensing yarns. The cracks formed on Ag/MXene/PU yarn and MXene/PU yarn are similar; however, the existence of Ag nanoparticles can effectively improve the conductivity even if the yarn is in a stretching state. Therefore, the signal intensity is better than that of Mxene/PU yarn. Due to the protection of the PDMS layer, it can be found that the cracks formed on the yarn after being coated with PDMS were narrower and fewer than those found in conductive yarns without PDMS coatings, indicating the excellent protective role of the PDMS layer and its contribution in improving the signal intensity.

The relationship between variation of resistance and applied strain on conductive yarns is presented in Figure S3. The variation of resistance increased with the increased strain. PDMS/Ag/MXene/PU yarn shows the largest resistance variation, while the other two conductive yarns are similar and far smaller than that of PDMS-encapsulated conductive yarn. This phenomenon also confirms the advantages of adding an elastic protective layer that cannot only protect the conductive layers, but also improves the working range. For the conductive yarns without a PDMS protective layer, the working range is limited to 100%, because the conductivity is destroyed completely after applying a strain over 100%. Gauge factor (GF) is an important parameter to evaluate the strain-sensing yarn, which can be calculated from Equation (2) [23]:

(2) GF=ΔRR0ε

where Δ*R* stands for the variation of resistance with a certain applied strain, *R*_0_ means the original resistance of conductive yarns, and *ε* stands for the applied strain. According to the equation, the GFs of different conductive yarns under different working ranges were calculated and are presented in Figure 4b. GFs calculated here were also used to evaluate the sensitivity of prepared strain-sensing yarns. It can be found that the yarn coated with a PDMS protective layer shows excellent sensing performance with an enlarged working range to 200%, while the other two are limited to 100%. Further, the signal response under different tensile ranges (25%, 50%, 75%, 100%, 125% and 150%) was investigated using PDMS/Ag/MXene/PU yarn, shown in Figure 4c. The cracks formed under the same strain were almost the same during the stretching/releasing process; therefore, the resistance variation basically remained a small fluctuation under the same strain. This result indicates that the prepared strain-sensing yarn shows a stable and continuous signal response to the cycling process. Meanwhile, the peak value of varied resistance increased continuously with the increasing strain degree. This performance ensured that the as-prepared sensing yarn exhibited the ability to monitor or distinguish different movements with different strains.

Moreover, the durability of the prepared conductive yarns was also evaluated and shown in Figure 4d–f. The yarns were tested with a 50% tensile strain. Among all the prepared conductive yarns, PDMS/Ag/MXene/PU yarn seems to be the most stable sensing yarn, with the minimum difference between the first cycle and the last cycle over 15,000 cycles, while Ag/MXene/PU yarn can run over 12,000 cycles with a slight fluctuation, and MXene/PU yarn shows a larger fluctuation and poor cycling stability. The results reveal that the conductive yarn coated with Ag nanoparticles and encapsulated with PDMS can effectively improve the conductivity and stability.

### 3.4. Applications

**Figure 5:**
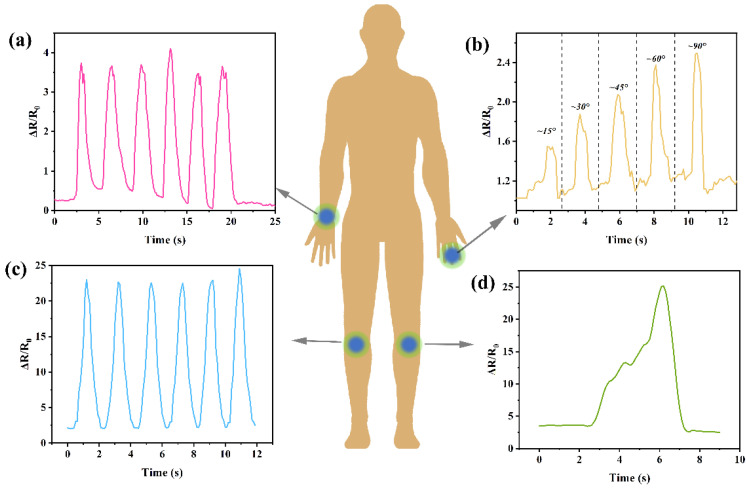
Applications for human motion monitoring: (**a**) bending the wrist, (**b**) bending fingers with different angles, (**c**) walking monitoring, and (**d**) slowly bending the knee.

Based on the performance evaluation, the prepared strain-sensing yarn (PDMS/Ag/MXene/PU yarn) was applied to monitor human motion. Figure 5a shows the signals detected during the bending of the wrist. It can be found that the signal and frequency can be easily obtained via the recorded curves. Moreover, the yarn was also fixed on the finger of the volunteer to check if it can be qualified for detecting minor changes. Here, the volunteer demonstrated different bending angles of his finger. It was found that the relative resistance (Δ*R*/*R*_0_) rapidly increased with the increased bending angles. This reveals that the prepared strain-sensing yarn shows the capability of application in calling devices, especially in those for bedridden inpatients. Figure 5c illustrates the obtained signals by monitoring the bending knee during the walking process. Compared with bending the wrist and finger, the relative resistance detected during the walking process is far higher; this indicates that the strain-sensing yarn can also be applied in monitoring movements with large amplitude, which is consistent with the basic analysis of the sensing yarn. Furthermore, a single signal of bending the knee slowly was also detected and shown in Figure 5d. It can be found that the shake of the knee during the bending process can also be clearly detected, indicating its excellent performance in monitoring human motion. All the results indicate that the prepared strain-sensing yarn shows excellent potential in effective real-time monitoring of different human motions, including walking, bending the wrist, bending the finger, or bending the knees, as well as small shakes during the bending actions. In the future, the prepared yarn may be combined with other technologies to achieve more functions.

## 4. Conclusions

In the current work, the strain-sensing yarn was successfully fabricated via the dip-coating method with PU yarn as elastic substrate and MXene nanosheets as conductive material. To improve its conductivity and stability, magnetron sputtering and PDMS encapsulation methods were introduced. The prepared strain-sensing yarn showed a working range of 200%, excellent stability over 15,000 cycles, and high sensitivity with a GF value over 700 under the strain region of 150–200%, as well as fast responsibility. The results also concluded that the sensing yarn showed excellent potentials in human motion monitoring, no matter the tiny motion or large-scale motions of human joints. There may be a great potential for such strain-sensing yarns to play a role in healthcare or smart wearable devices in the future.

## Data Availability

Not applicable.

## Supplementary Materials

The following supporting information can be downloaded at: https://www.mdpi.com/article/10.3390/polym14245401/s1, Table S1: Conductivity of different samples; Figure S1: Optical image of prepared strain sensing yarn (left: before PDMS coating, right: after PDMS coating); Figure S2: Stretching/releasing paths of different conductive yarns; Figure S3: Relationship between variation of resistance and applied tensile strain.
