# Supplementary material for: PDMS/Ag/Mxene/Polyurethane Conductive Yarn as a Highly Reliable and Stretchable Strain Sensor for Human Motion Monitoring
Source: Polymers (Basel). 2022 Dec 9;14(24):5401. doi: 10.3390/polym14245401 (PMC9783540; doi:10.3390/polym14245401)
Supplement: Supplementary file 1 [file polymers-14-05401-s001.zip › polymers-2044359-supplementary.pdf]

polymers

Electronic Supporting Information

PDMS/Ag/Mxene/polyurethane conductive yarn as high reliable and stretchable strain sensor for human motion monitoring

Zhang Shichen [1,*], Xu Jiangtao [2]

1. School of Innovation Design, Guangzhou Academy of Fine Arts, Guangzhou 510006, China

2. School of Fashion & Textiles, The Hong Kong Polytechnic University, Hong Kong, China

[*] Correspondence: shichenzhang@gzarts.edu.cn

Table S1 Conductivity of different samples

Samples	Conductivity (ms/cm)
MXene/PU yarn (without pretreatment)	N.A.
MXene/PU yarn (with piranha solution treatment only)	47.6
MXene/PU yarn (with CTAB treatment only)	61.8
MXene/PU yarn (with all treatment)	112
Ag/MXene/PU yarn	441.3

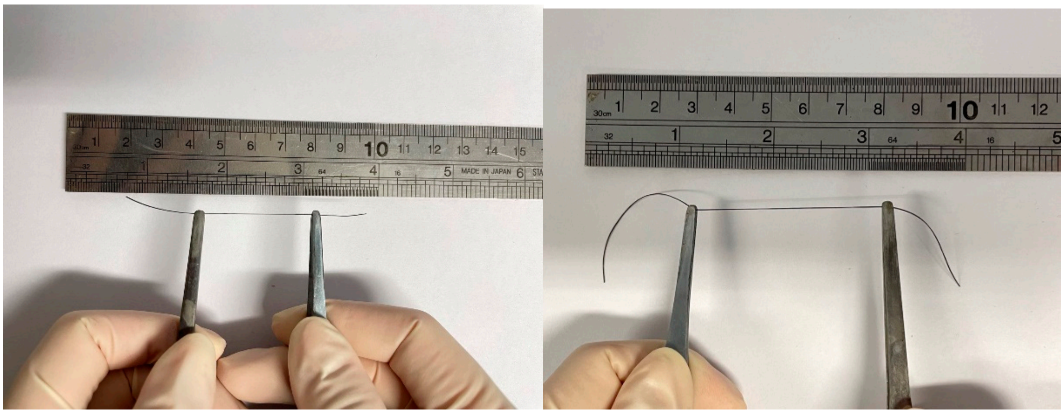

Figure S1 Optical image of prepared strain sensing yarn (left: before PDMS coating, right: after PDMS coating)

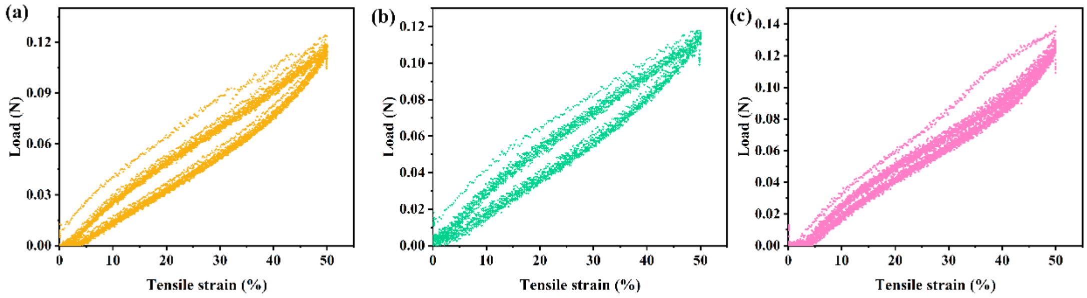

Figure S2 Stretching/releasing paths of different conductive yarns

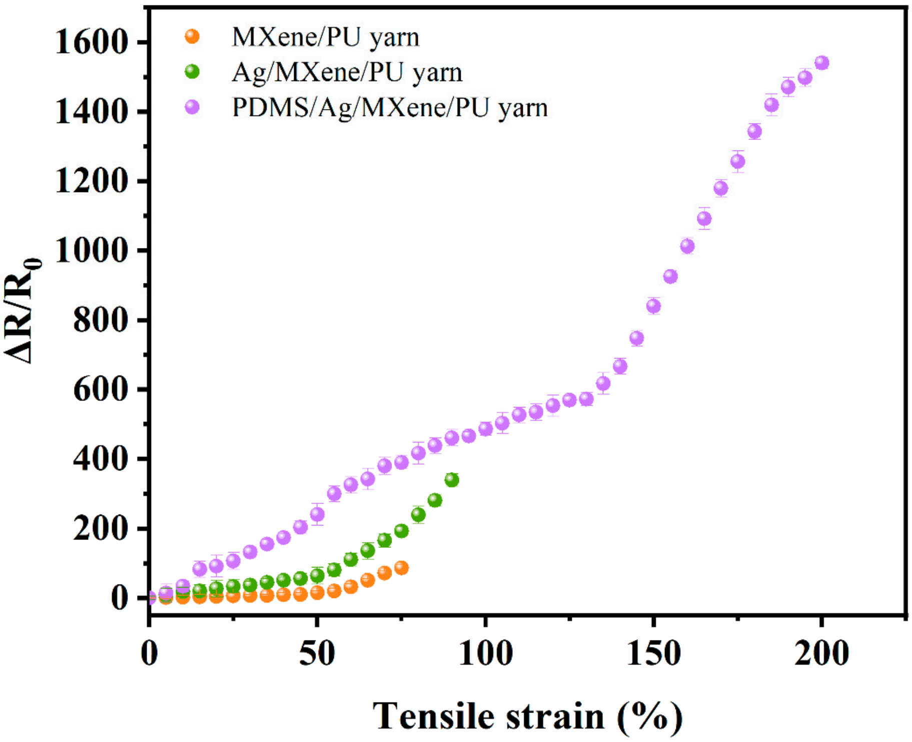

Figure S3 Relationship between variation of resistance and applied tensile strain
